# Measurement properties of physical function scales validated for use in patients with rheumatoid arthritis: A systematic review of the literature

**DOI:** 10.1186/1477-7525-9-99

**Published:** 2011-11-07

**Authors:** Martijn AH Oude Voshaar, Peter M ten Klooster, Erik Taal, Mart AFJ van de Laar

**Affiliations:** 1 Arthritis Center Twente, University of Twente, Department of Psychology, Health and Technology, Enschede, The Netherlands; 2 Department of Rheumatology, Medisch Spectrum Twente, Enschede, The Netherlands

**Keywords:** Physical function, disability, rheumatoid arthritis, psychometric, validity, reliability, responsiveness, measurement properties

## Abstract

**Background:**

The aim of this study was to systematically review the content validity and measurement properties of all physical function (PF) scales which are currently validated for use with patients with rheumatoid arthritis (RA).

**Methods:**

Systematic literature searches were performed in the Scopus and PubMed databases to identify articles on the development or psychometric evaluation of PF scales for patients with RA. The content validity of included scales was evaluated by linking their items to the International Classification of Functioning Disability and Health (ICF). Furthermore, available evidence of the reliability, validity, responsiveness, and interpretability of the included scales was rated according to published quality criteria.

**Results:**

The search identified 26 questionnaires with PF scales. Ten questionnaires were rated to have adequate content validity. Construct validity, internal consistency, test-retest reliability and responsiveness was rated favourably for respectively 15, 11, 5, and 6 of the investigated scales. Information about the absolute measurement error and minimal important change scores were rarely reported.

**Conclusion:**

Based on this literature review, the disease-specificHAQ and the generic SF-36 can currently be most confidently recommended to measure PF in RA for most research purposes. The HAQ, however, was frequently associated with considerable ceiling effects while the SF-36 has limited content coverage. Alternative scales that might be better suited for specific research purposes are identified along with future directions for research.

## Background

Patients' assessment of physical function (PF) is a core outcome domain of disease status in rheumatoid arthritis (RA)[1,2]. Physical function scales are used in the majority of clinical trials to assess the effectiveness of treatment and have become established instruments for assessing health outcomes in clinical practice and observational studies as well [3-5].

A number of efforts have currently been undertaken to compare the variety of disease-specific and generic PF scales that have been validated for use in patients with RA over the years [6-11]. However, previous efforts have been limited to descriptive reviews of well-known instruments or non-systematic selections of the available literature on their measurement properties. To date, there are no comprehensive studies available that systematically evaluate the evidence for the quality of the measurement properties of all PF scales that are validated for patients with RA. Furthermore, until recently there was no comprehensive conceptual framework available to define physical function in RA and with which to judge the relevance and comprehensiveness of the items of PF scales. Therefore, content validity could only be evaluated indirectly in previous efforts, for example by evaluating whether patients were included in the item selection process. Currently, the International Classification of Functioning, disability and Health (ICF) provides a comprehensive frame of reference, which allows the relevance and comprehensiveness of the items of PF scales to be examined directly by linking them to their respective ICF codes. Within the ICF classification, the 'activity' dimension constitutesthe individual's perspective on functioning and is defined as 'difficulties an individual may have in executing activities [12]. This dimension consists of the chapters domestic life, self-care and mobility, which respectively coincide with (instrumental) activities of daily living (IADL & ADL) and mobility which are traditionally used terms in the literature on physical functioning [13].

The most relevant ICF categories for a particular condition are summarized in a core set. The ICF Core Set for RA is a list of the ICF categories, which represent the typical functional problems experienced by patients with RA [14]. The outcome measures in rheumatology (OMERACT) group accepts the ICF core set for RA as the best currently available external standard of functioning and recognizes its utility for assessing the content validity of existing measurement instruments [15].

The aim of this study was to systematically review the content validity and measurement properties of all PF scales that have been validated for use in patients with RA, by linking their content to the ICF and to appraise the currently available evidence of the quality of their measurement properties in order to offer recommendations for the use of PF scales for various purposes and settings.

## Methods

### Study selection

An extensive literature search was conducted to retrieve all relevant articles related to the psychometric evaluation of PF scales in RA. A validated and sensitive search strategy for finding studies on measurement properties of patient-reported outcomes (PROs) was followed to design the search strings [16] and applied to the Scopus (1972-2010) and PubMed databases (1975-2010) in January 2011. This search strategy consists of four sets of independent searches that are later merged. The first search contains various synonyms of the construct of interest (i.e., physical function). The second search contains search terms for the population of interest (i.e., RA patients). The third search contains the validated and sensitive filter for the identification of studies investigating measurement properties of health-related PROs and the fourth search contains an exclusion filter. For more details about the content of the filters we refer to Terwee et al [16].The full search strings used in PubMed are available from the corresponding author.

Two reviewers (MOV and PTK) independently screened the titles and abstracts of the search results to identify potentially relevant studies. Studies were eligible if they were published in English, the main focus of the article was the development or psychometric evaluation of a questionnaire, at least part of the study population consisted of patients with RA, and the questionnaire was intended for use in adults. Final decisions on inclusion of studies in the review were made by consensus after both reviewers read all full-text articles that were deemed potentially relevant by either reviewer individually.

Questionnaires were retained for further review if they contained at least one scale addressing an aspect of overall PF (i.e., the ability to carry out basic or instrumental activities of daily living or mobility tasks), and were not limited to assessing the functioning of specific joints or limbs. Given the difficulty of assessing the quality of the applied translation procedures and the equivalence of translated versions of the questionnaires, only studies examining the measurement properties of the original language version were included. In case the original language of a questionnaire is spoken as the majority language in other countries, studies from those countries were considered to have used the original version, unless stated otherwise in the article. Finally, because the quality criteria used in this study require at least 50 patients per analysis to be eligible for rating, studies were included if analyses were reported for at least 50 patients with RA [17]. Furthermore, in case patient groups with various diseases were studied that were not analysed per patient group, studies were included if the study population contained at least 50% patients with RA, as has been done in similar, previous systematic reviews [18].

To ensure that all relevant studies were retrieved, a second series of searches was performed with the names of the retained questionnaires as search terms in addition to the words "rheumatoid arthritis" and references of included studies and studies citing the original article were manually searched using Scopus citation tracker. Lastly, search results were verified against previous non-systematic review articles of PF scales [6-11].

The full name of each retained questionnaire, the year of its development, and the language it was developed in were extracted, as well as the names of all scales relevant to the assessment of PF and their respective number of items. The consensus based standards for the selection of health status measurement instruments (COSMIN) checklist [19] was used to identify and extract information on measurement properties that are considered relevant for PROs. The COSMIN checklist was developed in a Delphi study among 43 experts in the field of health outcome measurement and contains standards for which measurement properties are most relevant to HR-PROs and standards for how these measurement properties should be evaluated in terms of study design and statistical analysis. Two reviewers (MOV & PTK) independently scored the checklist according to instructions in the manual for all included studies. Consensus about the ratings was reached by discussion. The quality of the measurement properties was rated according to quality criteria that were proposed for the COSMIN checklist [17]. An overview of all data relevant to the rated measurement properties is available in the supplementary material (additional File 1, additional File 2 & Additional File 3.).

#### Validity

Validity refers to the degree to which a scale measures what it sets out to measure [20]. Since no gold standard exists for patient reported physical function, scales should demonstrate content and construct validity [21].

Content validity should be assessed by making judgments about the relevance and the comprehensiveness of the items for assessing physical functioning of patients with RA [19]. The relevance of a scale was rated positively if all items of a scale could be linked to ICF codes that are included in the ICF core set for RA and belong to one of the three chapters of the activity domain: self-care, domestic life or mobility. A scale was considered to measure PF comprehensively in case its content covers all three chapters of the activity dimension of the ICF. For this analysis all items of the included scales were linked to the ICF according to peer-reviewd linking rules [22].

Construct validity refers to the extent to which scores on a questionnaire relate to other measures in a manner that is consistent with theoretically derived hypotheses concerning the constructs that are measured [23]. However, in the included studies, hypotheses were rarely specified a priori when the construct validity of a scale was examined. This lack of hypotheses about the magnitude of expected relationships with clinical or other PROs limits interpretation of the results. Based on text book recommendations, included studies that did specify hypotheses and previous experience with validating PF scales, the following set of hypotheses was specified [24-33]: A PF scale with adequate construct validity should correlate most strongly with other PF instruments, it should correlate second most strongly with other patient-reported measures of physical aspects of health (e.g., pain or the physical component score of the SF-36). PRO measures of non-physical aspects of health and clinical outcome measures (e.g., tender and swollen joint counts) should be less strongly related to the PF scale than the previous measures. Finally, we would expect the least strong correlations with (biological) process measures of disease activity. With respect to the absolute magnitude of correlations, a valid measure of PF was expected to correlate strongly (*r *> 0.60) with other measures of PF and measures of other aspects of physical health and moderately (0.30 <*r *< 0.60) with clinical outcome measures and patient reported non-physical aspects of health. Following the quality criteria of Terwee et al for a positive rating for construct validity [17], at least 75% of hypotheses should be confirmed and, in case a scale was validated against other established (multi-item) self-reported measures of PF, we considered it to be vital that the correlation was strong (*r *> 0.60).

#### Internal consistency

Scales that are internally consistent are made up of items that all measure the same concept and consequently produce correlated scores. When correlations among items are too high, however, redundant content is indicated [17]. Questionnaires received a positive rating for internal consistency if factor analysis indicated the homogeneity of each relevant scale in a sufficiently large sample (≥5 patients for every item in the analysis) and Cronbach's α was ≥0.70, but ≤0.95 for each relevant scale or the person separation index (or person reliability) was ≥0.70 if Rasch analysis was applied [17].

#### Reproducibility

This concerns the degree to which repeated measurements in stable patients provide similar results. We assessed agreement and test-retest reliability. Studying agreement is important to detect systematic differences between measurements and to establish how much scores of individual patients can be expected to vary from one occasion to the next when there is no real change in functional status [34,35]. The standard error of measurement (SEM) or limits of agreement (LOA) [34] were considered to be adequate parameters of agreement. Agreement was considered acceptable if the minimal important change (MIC, see under interpretability) was greater than the smallest detectable change, which can be calculated from the SEM, or if the MIC was outside the LOA. Because the MIC was not commonly reported, we also gave a positive rating in case the authors provided convincing arguments that agreement was acceptable.

Scales that are reliable, reproducibly distinguish between patients with unchanged levels of PF, despite measurement error. A positive rating for test-retest reliability was given if the intraclass correlation coefficient (ICC) for continuous measures or weighted kappa for categorical measures was ≥0.70 in a sample of at least 50 stable patients over a period of one to six weeks [17].

#### Responsiveness

The ability of a questionnaire to detect clinically meaningful changes over time, even if those changes are small, is called responsiveness [36]. Measuring change over the course of a therapeutic intervention with known effectiveness was considered to be the most appropriate technique for assessing responsiveness of PF scales [37,38]. A positive rating was given when adequate statistics, such as the standardized effect size or the standardized response mean, indicated a treatment effect of at least 0.30, which constitutes a moderate magnitude according to Cohen [39]. Because observed treatment effects depend critically on contextual elements such as the treatment used, the disease severity of the study sample, and the employed time frame, an adequate description of these elements was required for a positive rating as well.

#### Interpretability

Finally, it is important that clinicians and policy makers are able to assign qualitative meaning to questionnaire scores. Three aspects of interpretability were given individual ratings. First, minimally important change (MIC) scores should be documented. The MIC is the smallest change in score perceived to be important. Given that PRO measurement is inherently about the patients' perspective and that there is no objective gold standard for adequate changes in functional status, anchor-based techniques where patients rated the amount of change they experienced on a transition question, were considered to be appropriate. A positive rating was given if an adequate external indicator was used to categorize patients according to change status, the indicators were adequately described, and the relationship of the indicator with the questionnaire was sufficiently documented [37].

Secondly, substantial floor and ceiling effects should be absent. A large percentage of patients at the floor or ceiling of a measure limits the interpretability of change scores because further deterioration or improvement in functional status may occur but cannot be detected by the scale. A positive rating was given when ≤15% of patients either scored the lowest or highest possible score [17].

Finally, presenting scale scores for relevant subgroups of patients or before and after treatment and relating questionnaire scores to other outcome measures facilitates interpretability. A positive rating was given if at least two of the following types of information were presented: means and standard deviations before and after treatment with proven effectiveness, differences in scores between relevant groups, relating scores to patient's global ratings of change in disability or presenting information on the relationship of scores to other well-known measures of disability.

## Results

### Selection of studies

The main search yielded a total of 3257 hits, of which 306 studies met the inclusion criteria and were retrieved for review. Of the 110 questionnaires that were psychometrically evaluated in the studies, 65 did not contain a (separate) PF scale and 18 questionnaires were limited to assessing the functioning of specific limbs or joints. The 51 studies identified by the main search that examined the measurement properties of the original language version of one of the 26 retained questionnaires were kept for review. Manual searching and reference checking resulted in the identification of 3 additional studies that were reviewed as well.

### Description of the questionnaires

Table 1 summarizes the characteristics of the included questionnaires. In case a questionnaire was originally developed for use in patient groups other than RA, the original article about the development of the questionnaire was consulted. For descriptive purposes, questionnaires were grouped as generic (7 questionnaires) in case they were developed for use in diverse or general populations or disease-specific (19 questionnaires) when the questionnaire was developed for use in arthritic populations, according to the original articles.

**Table 1:** Descriptive information of included questionnaires

Questionnaire	Year	Original language	Target population	Relevant scales (# of items)
Generic questionnaires				
BI	1955	English (US)	Chronic illnesses/Rehabilitation patients	Barthel Index (10)
GARS	1993	Dutch	Older patients	Activities of daily living (8), Instrumental activities of daily living (11)
MHIQ	1976	English (US)	Free living populations	Physical function index (24)
NHP	1980	English (UK)	General population	Physical Mobility (8)
SF-36	1992	English (US)	General population	Physical functioning (10)
SIP	1975	English (US)	General sick population	Ambulation (12), Body care and movement (23), Mobility (10)
WHODAS-II	1999	Multilingual	General population	Getting Around (5), Self-care (4), Life activities (8)
Diseases specific Questionnaires				
FSI	1980	English (US)	Osteoarthritis	Mobility (3), Personal care (4), Home chores (4), Hand activities (3)
AIMS	1979	English (US)	Arthritic conditions	Mobility (4), Physical activity (5), Activities of daily living (4), Dexterity (5)
Short AIMS	1991	English (US)	Arthritic conditions	Mobility (2), Physical activity (3), Activities of daily living (2), Dexterity (3), Household activities (4)
Shortened AIMS	1989	English (US)	Arthritic conditions	Mobility (2), Physical activity (2), Activities of daily Living (2), Dexterity (2), Household activities (2)
AIMS2	1991	English (US)	Arthritic conditions	Mobility (5), Walking and bending (5), Hand and finger function (5), Arm function (5), Self-care (4), Household (4)
AIMS2-SF	1993	French	Arthritic conditions	Physical component (12)
CSHQ-RA	2006	English (US)	Rheumatoid arthritis	Dexterity (7), Mobility (8)
CSHQ-RA, revised	2006	English (US)	Rheumatoid arthritis	Dexterity (6), Mobility (6)
CSSRD-FAS	1995	English (US)	Rheumatoid arthritis	Personal care (14). Mobility (1), Transfer (1) Work/play (18)
FFbH	1990	German	Polyarthritic conditions	Funktions fragenbogen (18)
HAQ	1980	English (US)	Arthritic conditions	Disability index (20)
HAQ-II	2004	English (US)	Arthritic conditions	Disability index (10)
MDHAQ (10-ADL)	1983	English (US)	Arthritic conditions	Disability index (10)
MDHAQ (14-ADL)	2005	English (US)	Arthritic conditions	Disability index (14)
MHAQ	1983	English (US)	Arthritic conditions	Disability index (8)
ROAD	2005	Italian	Early arthritis	Upper extremity function (5), Lower extremity function (4), Activities of daily living/work (3)
IRGL	1990	Dutch	Arthritic conditions	Mobility (7), Self-care (8)
TFCQ	1982	English (US)	Rheumatoid arthritis	Mobility (4), Personal care (4), Arm/hand functions (7), Work/play (4)
SIP-RA	1993	Swedish	Rheumatoid arthritis	Body care and movement (14), Mobility (5)

BI = Barthel Index, GARS = Groningen Activity Restriction Scale, MHIQ = McMaster Health Index Questionnaire, NHP = Nottingham Health Profile, SF-36 = MOS 36 item short form Health survey, WHODAS-II = World Health Organization Disability Schedule-II, FSI = Functional Status Index, AIMS = Arthritis Impact Measurement Scales, Short AIMS = Short Arthritis Impact Measurement Scales, Shortened AIMS = Shortened Arthritis Impact Measurement Scales, AIMS2 = Arthritis Impact Measurement Scales 2, CSHQ-RA = Cedars-Sinai Health Related Quality of Life for Rheumatoid Arthritis instrument, CSHQ-RA Revised = Cedars-Sinai Health Related Quality of Life for Rheumatoid Arthritis instrument Revised, CSSRD-FAS-FAS = Cooperative Systematic Studies for Rheumatic Diseases group Functional Assessment Survey, FFbH = Funktionsfragenbogen, Hannover, MDHAq = Multidimensional Health Assessment Questionnaire, M-HAQ = Modified Health Assessment Questionnaire, HAQ = Health Assessment Questionnaire, HAQ-II = Health Assessment Questionnaire II, ROAD = Recent Onset Arthritis Disability Questionnaire, SIP-RA = Sickness Impact Profile for Rheumatoid Arthritis, TFCQ = Toronto Functional Capacity Questionnaire IRGL = Impact van Reuma op Gezondheid en Leven.

### Measurement properties

Ratings of the measurement properties are presented in table 2. Each measurement property is qualified as adequate with good methodological quality (+), indeterminate because of doubtful methodological quality (0), or inadequate with good methodological quality (-), Question marks indicate insufficient information about an aspect.

**Table 2:** Content validity and measurement properties of included questionnaires

Questionnaire*	Relevance	Comprehen-siveness	Construct validity	Internal consistency	Test-retest reliability	Agree-ment	Respon-siveness	MIC	Ceiling/floor effects	Score distribution
*Generic scales*										
BI [56]	**-**	**-**	**-**	?	?	?	?	?	?	?
GARS [26,57]	+	+	+	+	?	?	0	?	?	?
MHIQ [58]	+	+	+	0	0	?	0	?	?	?
NHP [59-63]	+	-	+	?	0	?	0	?	?	+
SF-36 [64-69]	+	-	+	0	0	0	+	+	-	+
SIP [70-72]	-	-	**-**	?	0	?	0	?	?	+
WHODAS-II [73,74]	-	+	0	0†	0	?	0	?	-	?
*Disease-specific scales*										
FSI [75]	+	+	?	0†	0	?	?	?	?	?
AIMS [76,30,59-81]	-	+	+	-	0	?	+	?	?	?
Short AIMS [76]	+	+	+	0†	0	?	0	?	?	?
Shortened AIMS [77]	+	+	-	0†	0	?	?	?	?	?
AIMS2 [82]	+	+	+	+	0	?	?	?	?	?
AIMS2-SF [83]	+	-	+	0	+	?	+	?	?	?
CSHQ-RA [28,29,84]	-	+	+	+	+	?	0	+	?	+
CSHQ-RA, revised [27]	+	+	+	+	+	?	0	+	?	+
CSSRD-FAS [85]	-	+	+	?	-	?	?	?	?	?
FFbH [86]	+	-	+	0	?	?	0	?	+	?
HAQ [40,87,32,41,66,79-94]	+	+	+	+	+	0	+	+	-	+
HAQ-II [41]	+	+	+	0	?	?	0	?	+	?
MDHAQ (10-ADL) [40]	+	-	?	0	?	?	?	?	+	?
MDHAQ (14-ADL) [40]	+	+	?	0	?	?	?	?	+	?
MHAQ [40,41,93-95]	+	-	+	+	0	0	+	?	-	+
ROAD [31,96]	-	+	-	+	+	0	+	?	+	?
IRGL [97,98]	+	+	-	0	0	?	?	?	?	?
TFCQ [99]	?	?	?	0	?	?	?	?	?	?
SIP-RA [100]	-	-	0	0	?	?	0	?	?	?

+ = good measurement properties with adequate methodological quality; - poor measurement properties with adequate methodological quality; 0 = indeterminate quality of measurement properties because of inadequate methodological quality; ? = no information found. * For the full names of the questionnaires see legend of table 1. † No factor analysis was applied, but Cronbach's α < 0.70.

#### Content validity

In total, only 30 out of 591 (5%) concepts that were identified in the items could not be linked to the ICF. The vast majority of concepts were linked to the chapters Mobility (47%), Self-care (23%) or Domestic life (10%). Questionnaires were rated for relevance and comprehensiveness.

Of the generic questionnaires, the GARS, MHIQ, NHP and SF-36 were rated positively for relevance because all their PF items could be linked to one of the ICF chapters mobility, self-care or domestic life (see table 2). Three generic questionnaires were rated negatively for relevance. The BI and SIP contain items related to faecal and urinary incontinence (ICF codes B5253 and B6202), and an item about transferring oneself (D420), which is not part of the ICF core set for RA. The SIP also contains an item that was linked vestibular function of balance (B2351), which belongs to the domain body functions. The WHODAS-II contains an item that was linked to the general tasks and demands category (D2302) from chapter 2, general tasks and demands and an item linked to remunerative employment (D850).

Thirteen disease-specific questionnaires were rated positively for relevance because all their respective PF items could be linked to mobility, self-care or domestic life categories featuring in the core set. Five disease-specific questionnaires were rated negatively for relevance. SIP-RA contains an item that was linked to vestibular function of balance (B2351), which belongs to the domain body functions and an item linked to the category mobility of a single joint (B7101) from the body functions chapter. The CSHQ-RA contains an item linked to mobility of a single joint(B7101) as well and multiple items linked to sensory of pain (B280) in its dexterity and mobility scale and one item linked to sleep function (B134). The CSSRD-FAS contains an item linked to remunerative employment (D850). The AIMS contains an item related to carrying out daily routine (D2308) and the ROAD contains an item that was linked to basic interpersonal interactions (D710).

In the analysis of comprehensiveness, nine questionnaires, four of which generic, were rated negatively (see table 2). All negatively rated questionnaires lack items assessing the domestic life chapter of the ICF (i.e., IADL). Overall, only ten questionnaires received favorable ratings for both aspects of content validity, indicating that all their items are relevant to the assessment of PF of patients with RA and all three relevant ICF chapters are measured by the items making up the scale.

#### Construct validity

Of the included generic scales, the construct validity of the WHODAS-II could not be rated because only the construct validity of the total score was investigated, which also includes scales measuring non-physical aspects of quality of life. The MHIQ was rated favourably because it demonstrated adequate known-groups validity. The GARS, NHP and SF-36 were tested for convergent and/or divergent validity and given positive ratings because the results were in accordance with > 75% of hypotheses. The BI was rated negatively because it did not correlate strongly with the HAQ (*r *= 0.42) and the SIP was correlated only moderately to patient reported PF (*r *= 0.41).

For the disease specific scales, no information was available to rate the construct validity of the FSI, TFCQ and both versions of the MDHAQ. An indeterminate ratings was given to the SIP-RA because sub-scale scores were only being correlated to the total score. Eleven disease specific scales received a positive rating for construct validity. The AIMS2 and AIMS2-SF were rated favourably because respectively known-group comparisons and multitrait methods indicated adequate construct validity. The remaining nine scales received positive ratings because the pattern of correlations was in sufficient agreement with our hypotheses. Only the ROAD, IRGL and shortened AIMS were given negative ratings for construct validity. all of the subscales of the ROAD were found to be inadequately related to the HAQ (*r *= 0.17-0.32), and the SF-36 PF scale (*r *= 0.18-0.32). Furthermore, because the scales were generally weakly related to other measures relevant to our hypotheses (see supplementary material) eventually only 4 out of 25 (16%) hypotheses were confirmed. For the IRGL and the shortened AIMS, 65% and 51% of hypotheses were confirmed, respectively.

#### Internal consistency

For 11 out of 22 questionnaires for which information on internal consistency was found, factor analysis was applied before calculating Cronbach's α. The AIMS was the only questionnaire to receive a negative rating, because α < 0.70 was reported for the physical activities and activities of daily living subscales. The HAQ-II and SF-36 were rated indeterminate because internal consistency was tested with Rasch analysis and although the person separation index was deemed acceptable (≥0.70) in both cases, there was no assessment of the dimensionality of the scales beyond the reporting of item level fit statistics. The AIMS2-SF and both versions of the MDHAQ were rated indeterminate because a single scale was created for PF, while factor analysis had indicated the presence of multiple dimensions. Inappropriate statistical methods were used for the TFCQ, the sample size was < 50 patients for the MHIQ, and for the SIP-RA internal consistency analysis was performed on the total questionnaire scores only, rather than on individual scales, leading to indeterminate ratings for these questionnaires as well. For the remaining questionnaires that were rated indeterminate, factor analysis had not been applied.

#### Reproducibility

The HAQ, CSHQ-RA, revised CSHQ-RA, ROAD, and AIMS2-SF were rated positive for reliability. The NHP, AIMS, IRGL, and both of the AIMS short forms were rated indeterminate for reliability because the Pearson product moment correlation was used instead of the ICC. The SIP, MHIQ, WHODAS-II, SF-36, AIMS2, and MHAQ were rated indeterminate because the sample size was < 50. ICCs for individual items only were reported for the FSI. Only the CSSRD-FAS received a negative rating, because ICCs < 0.70 were observed for the transfer and mobility scales in stable patients.

The LOA or SEM was presented for only four questionnaires. For the ROAD, the limits of agreement were not related to the MIC, nor were arguments provided with respect to the acceptability of the level of agreement between scores on different times. For the HAQ, MHAQ, and SF-36, the SEM or LOA were estimated in a sample < 50 patients. Therefore, agreement was rated indeterminate for all scales.

#### Responsiveness

Information on responsiveness was found for 17 questionnaires. Six questionnaires were rated positive for responsiveness, because either the standardized effect size or the standardized response mean statistic showed moderate improvements in scores after effective treatment, with adequate descriptions of contextual factors. Studies on the GARS, WHODAS-II, and HAQ-II also utilized appropriate statistics, but their results couldn't be interpreted because insufficient information was presented about the study design or results. Methods that merely rely on the significance of the difference between scores at two time points were used for the CSHQ-RA, revised CSHQ-RA, TFCQ, and short AIMS. These statistical techniques were considered inadequate because p-values are inversely related to sample size. For the SIP and SIP-RA unconventional methods were used to examine its responsiveness. The remaining scales that were rated indeterminate had sample sizes < 50 patients.

#### Interpretability

MICs were reported for four questionnaires. Marked floor effects were reported for the SF-36, where 22% of a sample stratified to equally represent patients from all four Steinbrocker functional classes scored the worst possible score. However, this was caused almost exclusively by patients in Steinbrocker functional classes III and IV. Ceiling effects of up to 31% of patients were reported for the MHAQ, 16% for the HAQ, and > 15% for the WHODAS-II. For the remaining questionnaires that were rated, floor and ceiling effects were all well below the cut-off point of 15%. For seven questionnaires, two or more types of score distributions were presented that can facilitate the interpretation of questionnaire scores.

## Discussion

This study systematically reviewed the literature on measurement properties of PF scales that are validated for use in patients with RA. The results of this review provide a comprehensive assessment of the available evidence for the utility of available scales for patients with RA and may inform the appropriate selection of self-reported PF scales for various purposes in clinical practice and research.

PROs are commonly classified as disease-specific or generic. In this systematic review, a pragmatic classification was employed based on the intended target population of the included questionnaires. However, it should be noted that although developed for use in arthritic populations, PF scales that were classified as disease-specific do not necessarily have content that is exclusively relevant in these populations. In fact, some scales such as the HAQ which is often referred to as a disease-specific measure, assesses physical disability in general and does not focus on specific disease-associated impairments. As a result, the scale has been used across a wide range of general and clinical populations [3].

Of the disease-specific scales that were rated positively for both aspects of validity, the HAQ received the most favourable overall evaluation. Owing to its longstanding and extensive use in RA, the measurement properties of the HAQ have been exhaustively studied. This review showed that it has predominantly favourable measurement properties that have been studied with adequate methodological rigor. The HAQ met the standards we set for responsiveness and its test-retest reliability was found to be very high in a sample of stable patients, indicating that the scale is appropriate for evaluative purposes (i.e., to track physical functioning over time), both at the group level and at the individual level. However, one important limitation of the HAQ is that multiple studies noted a considerable group of patients scoring the best possible score. Therefore, it may not be the most appropriate scale for use in patient populations with relatively good functional capacity, since it cannot measure improvement in a substantial proportion of patients. Both the MDHAQ (14 ADL) and the HAQ-II were rated favorably for all aspects of validity as well and were specifically developed to address the ceiling effects of the original HAQ [40,41]. Both scales indeed demonstrated substantially smaller ceiling effects in direct comparison with the original HAQ, indicating that these scales might be more appropriate than the original HAQ for use in relatively well functioning groups. Another advantage of these scales is that they contain only 14 and 10 items, making them more feasible for use in clinical practice or when administering multiple PROs simultaneously. However, the measurement properties of HAQ-II and MDHAQ (14-ADL) have been less extensively studied. In particular, before recommending their use in evaluative studies, the responsiveness of these scales should be compared to that of the HAQ and their reproducibility in stable patients should be established. The revised CSHQ-RA and AIMS2 were also rated favorably for validity, but no information is available known about their distributional properties and the evidence testifying to the responsiveness of the revised CSHQ-RA is limited to methods that rely on statistical significance. Further research is required before a comprehensive evaluation of the quality of the revised CSHQ-RA is possible. The AIMS2 might be the most comprehensive disease-specific questionnaire. Its items were linked to 31 relevant ICF categories and issues such as fine hand use and arm use and domestic life are addressed in more detail than in the HAQ, which was also noted by Stucki et al [14]. However, with its 28 items it is also the most lengthy questionnaire and much of the work on its measurement properties is outdated. Further psychometric testing is therefore desirable. Finally, the short AIMS was also rated favorably for all aspect of validity, but it contains scales that lack internal consistency, perhaps because some subscales consist of only 2 items or because the response format is often yes/no. Therefore we would not recommend it for use or for further testing.

The CSHQ-RA and ROAD are among the most recently developed disease-specific scales and the methodology of the work on their measurement properties conforms to the rigorous methodological standards of COSMIN, enhancing the interpretability of their psychometric quality in this review. Regrettably however, these scales suffer from irrelevant content. Therefore their use cannot be recommended for the assessment of PF, despite generally favorable evaluations for their other measurement properties.

Although it is well known that measurement properties are context-specific attributes that can differ across populations, previous studies have paid no attention to verifying the content validity of the included generic scales for use in RA patient groups. Therefore, by linking their content to the comprehensive ICF core set for RA, this review provides the first assessment of the content validity of included generic scales for assessing physical functioning of patients with RA.

The SF-36 PF scale is probably the most frequently used generic scale in patients with RA. However, although all of its items are relevant, it measures predominantly mobility and has no content relevant to the assessment of domestic life, which was already recognized as an important shortcoming by its developers [42]. Another limitation of the scale is that it has been associated with substantial floor effects (i.e., patients scoring the worst possible score). Most of its measurement properties have been studied in patients with RA, but studies of more rigorous methodological quality are desirable. For instance, no studies were found reporting on the dimensionality of the original version and its reproducibility has been studied in small patient groups (n < 25) only. On the other hand, the SF-36 PF-10 is the only generic PF scale that was rated positively for responsiveness.

Except for the MHIQ, the other health profiles, (SIP and NHP) demonstrated limited content coverage as well. Because health profiles intend to cover all major areas of health, it might be expected that content coverage within their components is less comprehensive. The GARS on the other hand is a dedicated PF instrument which is reflected in the finding that its content more comprehensively reflects the overall PF domain. Therefore, the GARS may be well suited when the primary outcome of interest is physical function rather than overall health. However, as with most generic scales in this review, its measurement properties are currently poorly understood. More research is required to establish its performance in longitudinal settings before its use can be recommended.

With the inclusion of items of the participation chapters of the ICF, the WHODAS-II covers a wider spectrum of disability than just physical function. The same applies to the BI and SIP. These measures include multiple items belonging to ICF categories E120 (Products and technology for personal use in daily living), E30 (support and relationships) and B5253 and B6202 (fecal/urinary incontinence). Therefore, they might be better thought of as measures of dependence rather than physical function per se. This interpretation is further strengthened by the observation that the SIP and BI were evaluated negatively for construct validity. In particular, both scales correlated only moderately with other PF instruments.

With respect to rating the measurement properties of the included scales, it was notable that in one-third of the studies that assessed reliability, samples of less than 25 patients were used. Although observed ICCs were generally well above the commonly accepted cut-off point of 0.70, it is important that reliability is studied in sufficiently large samples. Simulation studies have shown that even when a value as high as 0.80 is observed, a sample size of 60 patients is required to reliably conclude that ICC > 0.70 in the population the sample was drawn from [43,44]. Furthermore, for most scales, information on reproducibility of scores was limited to reports on test-retest reliability. For evaluative purposes, especially when monitoring functional status of individual patients, it is informative to report on the absolute agreement of test-retest scores for patients with unchanged functional status as well. Representative values of the LOA or SEM can serve as benchmarks for distinguishing real change in functional status from changes due to random measurement error [17]. Finally, minimally important change scores have not been widely reported and should be addressed in future research, as they greatly enhance the interpretability of change scores. Instruments should be administered longitudinally before and after treatment known to improve PF, and health transition questions should be included as external criteria of change (26). A point worth mentioning is that this systematic review is limited to traditional static questionnaires.

Recently, item response theory (IRT) based item banking is receiving increasing attention in PRO assessment. Of special relevance to PF assessment in RA populations is the patient reported outcome measurement information system (PROMIS) initiative. PROMIS is an NIH initiative aimed at revising instruments in many domains including PF, using IRT calibrations and computerized adaptive testing (CAT) [45]. The PROMIS PF item bank contains 124 calibrated items and CAT algorithms allow for the adaptive selection of the most relevant item for a particular patient in terms of relative difficulty based on previous answers given by that patient [46]. The main advantage of using these modern psychometric approaches is that the use of extensive item banks potentially eliminates floor and ceiling effects, while the CAT algorithm ensures that patients only need to answer a minimum number of questions [47,48]. Short forms can also be developed from the PROMIS item banks. For example, the PROMIS HAQ has been developed from the PROMIS PF item bank [46]. Unfortunately, none of the PROMIS studies met the inclusion criteria for this review of at least 50% RA patients, however the PROMIS PF item bank is likely to become a prominent measurement system in RA and it would be highly interesting for future research to study the psychometric properties of the PROMIS PF item bank specifically for RA populations.

There are some limitations to our study that deserve attention. First, we used the ICF as an external standard to evaluate the content validity of the included scales, as have a number of previous similar systematic reviews [49,50]. The ICF aims to provide a common language for functional status assessment in clinical practice and research. However, most included scales were developed before the ICF was available. Moreover, concerns have been voiced regarding the exhaustiveness of the ICF as a comprehensive classification of disability [51] and several validation studies of the ICF core set for RA have found some omissions from the perspective of patients and physicians that future research should address [52,53]. Therefore some caution must be taken when interpreting the results of the analysis of content validity. Still, the ICF is frequently recommended for assessing the content validity of health status instruments [15] and 95% of all PF items included in this systematic review could be linked to at least one ICF code. Moreover, the items that were linked to ICF categories other than mobility, self-care or domestic life were all clearly irrelevant to the assessment of PF. Our results therefore seem to indicate that the ICF is a useful taxonomic tool for assessing the relevance of disability items, such as those included in this systematic review. Second, for most scales, the work on their measurement properties was predominantly or exclusively performed with the original language versions. However, the majority of the studies on the measurement properties of the AIMS2 and AIMS2-SF concerned translated versions. Users of translated versions are therefore advised to examine if a validation study is available for their language version, rather than solely depending on the results of this review. For several translations, individual items were omitted, changed, or added in order to adapt a questionnaire for use in a different culture. Since in some instances up to 10% of items were changed, it is unclear to what degree measurement properties are generalizable across versions and cultures [54,55].

## Conclusions

None of the scales met all the rigorous quality requirements we set. However the disease-specific HAQ can confidently be recommended for most applications in patients with RA. Longitudinal or experimental studies in patient groups with relatively good functional capacity may require scales with broader measurement range such as the MDHAQ (14 ADL) or HAQ-II. However, since their longitudinal performance is currently poorly documented, their test-retest reliability and responsiveness should be addressed by future research first. The SF-36 is the most thoroughly evaluated generic scale that is currently most suited for studies that want to compare RA patients with other populations. In particular, it is the only generic scale with adequate proven responsiveness. However it has limited coverage of the PF domain and therefore it would be desirable to compare its performance in longitudinal settings with that of the GARS and MHIQ, which more comprehensively measure PF.

## Abbreviations

PF: physical function; RA: rheumatoid arthritis; PRO: patient reported outcome measures ICF: International classification of functioning disability and health; ADL: activities of daily living; IADL instrumental activities of daily living; OMERACT: outcome measures in rheumatology; COSMIN: The consensus based standards for the selection of health status measurement instruments; SEM: standard error of measurement; LOA: limits of agreement; ICC: intraclass coefficient; MIC: minimally important change score.

## Competing interests

The authors declare that they have no competing interests.

## Authors' contributions

MOV was responsible for the search strategy and conceptualisation of the manuscript. MOV and PTK reviewed the included papers. PTK, ET and MVDL supervised the study and the interpretation of the results. All authors critically reviewed, contributed to and approved the final manuscript.

## Supplementary Material

Additional file 1 **Supplementary table 1 validity.doc**. Click here for file

Additional file 2 **Supplementary table 2: reproducibility.doc**. Click here for file

Additional file 3 **Supplementary table 3 responsiveness and interpretability.doc**. Click here for file
