# Supplementary material for: Measurement properties of physical function scales validated for use in patients with rheumatoid arthritis: A systematic review of the literature
Source: Health Qual Life Outcomes. 2011 Nov 7;9:99. doi: 10.1186/1477-7525-9-99 (PMC3221621; doi:10.1186/1477-7525-9-99)
Supplement: Additional file 1 — Supplementary table 1 validity.doc. [file 1477-7525-9-99-S1.DOC]

| Scale | Ref. | Dimensionality  Studied? | α | Hypotheses? | Results | N |
| --- | --- | --- | --- | --- | --- | --- |
| MHIQ  Physical function index |  | No | 0.76 | No | Known groups validity  RAI:  0-19 = 0.42  20-29 = 0.29  30-39 = 0.25  40-59 = 0.23  Morning stiffness:  No = 0.39  Slight = 0.34  Moderate = 0.36  Severe = 0.25  Very severe = 0.21  Duration of morning stiffness:  30 min = 0.41  > 30 min = 0.31  ESR:  3-17 = 0.38  18 – 40 = 0.35  41-70 = 0.35  71-120 = 0.32 | 40 |
| GARS  Activities of daily living |  | Yes | 0.88 | No | Known groups validity  Steinbrocker functional classes:  I= 0.06,  II=0.45,  III=1.12 | 634 |
|  |  | Yes | ? | Yes | Convergent/divergent validity  R KPSS = 0.64  R OEH = -0.39  R NHP-PM = 0.77  R GHQ-SS = 0.26 | 630 |
| GARS  Instrumental activities of daily living |  | Yes | 0.88 | No | Known groups validity  Steinbrocker functional classes:  I = 0.24  II = 0.67  III = 1.89 | 634 |
|  |  | Yes | ? | Yes | Convergent/divergent validity  R KPSS = 0.66  R OEH = -0.40  R NHP-PM = 0.71  R GHQ-SS = 0.21 | 630 |
| WHODAS-II Getting around |  | No | 0.89 | Yes | * | 172 |
|  |  | No | 0.65 | Yes | * | 85 |
| WHODAS-II Self care |  | No | 0.82 | Yes | * | 172 |
|  |  | No | 0.79 | Yes | * | 85 |
| WHODAS-II Life activities |  | No | 0.90 | Yes | * | 172 |
|  |  | No | 0.84 | Yes | * | 85 |
| BI |  | No | ? | No | Convergent/divergent validity  R HAQ = 0.89 | 33 |
| SIP  Physical dimension |  | No | ? | No | Convergent/divergent validity:  R disease duration = 0.36  R morning stiffness = 0.15  R grip strength = -0.33  R Hematocrit = -0.26  R ESR = 0.44  R Anatomic stage = 0.31  R Employment status = -0.4  R Evidence of mental health problems = -0.03 | 79  55  20  65  39  72  49  79 |
|  |  | No | ? | No | Known group validity  ARA functional class:  I = 6.4,  II = 13.1  III = 19.3  IV = 37.3. |  |
|  |  | No | ? | N/A |  |  |
| SF-36 Physical functioning |  | No | ? |  |  |  |
|  |  | No | ? | N/A |  |  |
|  |  | No | ? | No | Convergent/divergent validity  R ESR = -0.40  R Joint tenderness = -0.48  R joint swelling = -0.42  R physician rated disease activity = -0.37  R patient rated disease activity = -040  R HAQ = -0.89  R VAS-pain = -0.56  R HAD = -0.44 | 233 |
|  |  | No | 0.92 | Yes | Known groups validity (change scores)  Patient global:  Improve 3+ levels: 13.5  Improve 2 levels: 16.1  Improve 1 level: 8.4  Same/worse: 3.2  Physician global  Improve 3+ levels: 20.5  Improve 2 levels: 13.2  Improve 1 level: 8  Same/worse: 4.1  Pain assessment  Improve 3+ levels: 13.7  Improve 2 levels: 6.4  Improve 1 level: 8.2  Same/worse: 4.5  SJC  Improve 3+ levels: 8.3  Improve 2 levels: 13.3  Improve 1 level: 6.4  Same/worse: 5.7  TJC  Improve 3+ levels: 10.8  Improve 2 levels: 8.5  Improve 1 level: 7.7  Same/worse: 3.9 | 693 |
|  |  | No | ? | No | Convergent/divergent validity  R ESR = -0.37  R CRP = -0.42  R Tender joints = -0.46  R Swollen joints = -0.25  R VAS-pain = -0.39  R Patient’s GA = -0.51  R Physician GA = -0.52  R HAQ = -0.75 | 86 |
|  |  | No | Person reliability = 0.88 | N/A |  |  |
|  |  | No | ? | No | Multitrait scaling analysis with M-HAQ and SF-36 Bodily pain scale. | 207 |
| NHP  Physical mobility |  | No | ? | N/A |  |  |
|  |  | No | ? | No | Convergent/divergent validity  R tender joint count = 0.47  R swollen joint count = 0.42  R VAS pain = 0.49  R Patient GA = 0.50  R Morning stiffness = 0.33  R DAS = 0.48  R ESR = 0.14  R C-reactive protein = 0.29  R Rheumatoid factor= 0.15  R HAQ = 0.82  R Larsen score = 0.26 | 200 |
|  |  | No | ? | No | Convergent/divergent validity  R Morning stiffness 0.50  R RAI = 0.34  R Grip = 0.39  R pain = 0.43 |  |
|  |  | No | ? | No | Known groups validity  Ra patients = 64.0  Migraine sufferers = 2.0 | 82 |
|  |  | No | ? | N/A |  |  |
| Disease specific scales |  |  |  |  |  |  |
| CSSRD Personal care |  | No | ? | No | Convergent/divergent validity  R Steinbrocker functional class =  >0.30, <0.57  R Clinical measures <0.59, exact data not shown | 110 |
| CSSRD, Mobility |  | No | ? | No | Convergent/divergent validity  R Steinbrocker functional class =0.30  R Clinical measures <0.59, exact data not shown | 110 |
| CSSRD Transfer |  | No | ? | No | Convergent/divergent validity:  R Steinbrocker functional class = >0.30, <0.57  R Clinical measures <0.59, exact data not shown | 110 |
| CSSRD Work/chores |  | No | ? | No | Convergent/divergent validity:  R Steinbrocker functional class =0.57  R Clinical measures <0.59, exact data not shown | 110 |
| HAQ-II |  | No | Person reliability = 0.88 | No | Convergent/divergent validity  R HAQ = 0.92  R M-HAQ = 0.85  R VAS-pain = 0.66  R patient assessment of global disease severity = 0.62  R VAS Fatigue = 0.57  R DAS28 = 0.51  R Physician’s global assessment of disease severity = 0.48  R stopped working = 0.41  R TJC = 0.37  R ESR = 0.25  R SJC = 0.24  R Joint surgery (Yes/no) = 0.20  R HAQ = 0.91  R Sf-36 physical functioning = -0.86  R M-HAQ = 0.85  R euroqol = -0.68  RADAI = 0.66  R Rheumatology distress index = 0.62  R Vas Pain = 0.62  R VAS global disease severity = 0.61  R SF-6D = -0.57  R VAS fatigue = 0.57  R Work limitations questionnaire = 0.55  R QOL scale = -0.54  R AIMS depression scale = 0.42  R Sleep disturbance = 0.42  R AIMS anxiety scale = 0.39  R Social security disability = 0.34  R GI severity = 0.33  R Total direct medical costs = 0.24  R Total joint replacement = 0.20 | 693  10.916 |
| FFbH |  | No | 0.94 | No | Convergent/divergent validity  R HAQ = -0.87  R Barthel = 0.61  R DAS 28 = >0.45, < 0.55 | 97 |
| CSHQ-Ra revised Dexterity |  | Yes | 0.95 | Yes | Known groups validity  HAQ score ranges  0-0.5 = 24.50  0.5-1.0 = 34.10  1.0-1.5 = 43.80  1.5-2.0 = 56.30  2.0-2.5 = 66.50  2.5-3.0 = 82.00  Convergent/divergent validity  R PCS = -0.58  R MCS = -0.43  R HAQ = 0.74 | 20  35  60  77  69  24  207 |
| CSHQ-Ra revised Mobility |  | Yes | 0.91 | Yes | Known groups validity  HAQ score ranges  0-0.5 = 32.50  0.5-1.0 = 47.90  1.0-1.5 = 54.80  1.5-2.0 = 66.10  2.0-2.5 = 76.30  2.5-3.0 = 89.40  Convergent/divergent validity  R PCS = -0.670  R MCS = -0.470  R HAQ = 0.720 | 20  35  60  77  69  24  207 |
| CSHQ-RA Dexterity |  | No | ? | Yes | Known groups validity  HAQ score range:  0-0.5 = 11.90  0.5-1.0 = 27.20  1.0-1.5 = 40.83  1.5-2.0 = 49.22  2.0-2.5 = 63.5  2.5-3.0 = 79.29  Convergent/divergent validity  R PCS = -0.55  R MCS = -0.35  R HAQ = 0.80 | 123  70  40  23  9  5  276 |
|  |  | No | 0.91 | Yes | Known groups validity  HAQ score ranges  0-0.5 = 27.90  0.5-1.0 = 39.40  1.0-1.5 = 47.40  1.5-2.0 = 59.50  2.0-2.5 = 69.20  2.5-3.0 = 82.80  Convergent/divergent validity  R PCS = -0.61  R MCS = -0.49  R HAQ = 0.76 | 20  35  60  77  69  24  207 |
|  |  | Yes | 0.89 | N/A |  | 291 |
| CSHQ-Ra  Mobility |  | No | ? | Yes | Known groups validity  HAQ score ranges  0-0.5 = 25.10  0.5-1.0 = 47.9  1.0-1.5 = 54.8  1.5-2.0 = 66.1  2.0-2.5 = 76.3  2.5-3.0 = 89.4  Convergent/divergent validity  R PCS = -0.78  R MCS = -0.38  R HAQ = 0.80 | 123  70  40  23  9  5  276 |
|  |  | No | 0.90 | Yes | Known groups validity  HAQ score ranges  0-0.5 = 39.90  0.5-1.0 = 53.50  1.0-1.5 = 59.90  1.5-2.0 = 68.80  2.0-2.5 = 78.50  2.5-3.0 = 87.80  Convergent/divergent validity  R PCS = -0.66  R MCS = -0.52  R HAQ = 0.69 | 20  35  60  77  69  24  207 |
|  |  | Yes | 0.92 | N/A |  | 291 |
| AIMS  Dexterity |  | Yes | 0.63 | No | Convergent/divergent validity  R Functional class = 0.37  R disease activity = 0.42  R walking time = 0.12  R grip strength = 0.46  R joint count = 0.38  R range of motion = 0.27 | 444  114 |
|  |  | No | ? | Yes | Convergent/divergent validity  R preference to obtain pain relief = -0.03  R preference to discuss emotional concerns = -0.08  R preference to obtain assistive devices = 0.26  R preference to increase ability to get around = 0.06 | 120 |
|  |  | No | 0.76 | No | Convergent/divergent validity  R joint count = 0.37  R joint alignment and motion scale = 0.44 | 180 |
|  |  | No | ? | N/A |  |  |
|  |  | No | 0.83 | No | Convergent/divergent validity  R VAS-pain = 0.45  R AHI helplessness = 0.35  R CES depression = 0.37  R AIMS global rating = 0.41  R Psychosocial impairment scale = 0.34  R activity limitation scale = 0.48  R VPMI active coping scale = -0.05  R VPMI passive coping scale = 0.19 | 369 |
|  |  | No | ? | N/A |  |  |
|  |  | No | ? | N/A |  |  |
| AIMS  Household activity |  | Yes | 0.81 |  | Convergent/divergent validity  R Functional class = 0.48  R disease activity = 0.28  R walking time = 0.26  R grip strength = 0.26  R joint count = 0.22  R range of motion = 0.20 | 444  114 |
|  |  | No | ? | N/A | Convergent/divergent validity  R preference to obtain pain relief = 0.13  R preference to discuss emotional concerns = 0.10  R preference to obtain assistive devices = 0.08  R preference to increase ability to get around = 0.20 | 120 |
|  |  | No | 0.81 | No | Convergent/divergent validity  R joint count = 0.41  R joint alignment and motion scale = 0.45 | 180 |
|  |  | No | ? | N/A |  |  |
|  |  | No | 0.74 | No | Convergent/divergent validity  R VAS-pain = 0.38  R AHI helplessness = 0.39  R CES depression = 0.39  R AIMS global rating = 0.42  R Psychosocial impairment scale = 0.34  R activity limitation scale = 0.52  R VPMI active coping scale = -0.21  R VPMI passive coping scale = 0.32 | 369 |
|  |  | No | ? | N/A |  |  |
|  |  | No | ? | N/A |  |  |
| AIMS  Activities of daily living |  | Yes | 0.71 |  | Convergent/divergent validity  R Functional class = 0.43  R disease activity = 0.37  R walking time = 0.27  R grip strength = 0.21  R joint count = 0.28  R range of motion = 0.27 | 444  114 |
|  |  |  |  |  | Convergent/divergent validity  R preference to obtain pain relief = 0.10  R preference to discuss emotional concerns = 0.12  R preference to obtain assistive devices = -0.04  R preference to increase ability to get around = -0.08 | 120 |
|  |  | No | 0.60 | No | Convergent/divergent validity  R joint count = 0.38  R joint alignment and motion scale = 0.35 | 180 |
|  |  | No | ? |  |  |  |
|  |  | No | 0.62 | No | Convergent/divergent validity  R VAS-pain = 0.46  R AHI helplessness = 0.32  R CES depression = 0.40  R AIMS global rating = 0.40  R Psychosocial impairment scale = 0.30  R activity limitation scale = 0.37  R VPMI active coping scale = -0.13  R VPMI passive coping scale = 0.35 | 369 |
|  |  | No | ? | N/A |  |  |
|  |  | No | ? | N/A |  |  |
| AIMS  Physical activity |  | Yes | 0.63 | No | Convergent/divergent validity  R Functional class = 0.52  R disease activity = 0.42  R walking time = 0.48  R grip strength = 0.29  R joint count = 0.40  R range of motion = 0.28 | 444  114 |
|  |  | No | ? | No | Convergent/divergent validity  R preference to obtain pain relief = 0.08  R preference to discuss emotional concerns = 0.06  R preference to obtain assistive devices = 0.10  R preference to increase ability to get around = 0.25 | 120 |
|  |  | No | 0.68 | No | Convergent/divergent validity  R joint count = 0.36  R joint alignment and motion scale = 0.41 | 180 |
|  |  | No | ? | N/A |  |  |
|  |  | No | 0.62 | No | Convergent/divergent validity  R VAS-pain = 0.50  R AHI helplessness = 0.43  R CES depression = 0.49  R AIMS global rating = 0.52  R Psychosocial impairment scale = 0.45  R activity limitation scale = 0.62  R VPMI active coping scale = -0.13  R VPMI passive coping scale = 0.23 | 369 |
|  |  | No | ? | N/A |  |  |
|  |  | No | ? | N/A |  |  |
| AIMS  Mobility |  | Yes | 0.85 |  | Convergent/divergent validity  R Functional class = 0.47  R disease activity = 0.28  R walking time = 0.46  R grip strength = 0.36  R joint count = 0.25  R range of motion = 0.18 | 444  114 |
|  |  |  |  |  | Convergent/divergent validity  R preference to obtain pain relief = 0.15  R preference to discuss emotional concerns = 0.01  R preference to obtain assistive devices = -0.08  R preference to increase ability to get around = 0.13 | 120 |
|  |  | No | 0.80 | No | Convergent/divergent validity  R joint count = 0.24  R joint alignment and motion scale = 0.31 | 180 |
|  |  | No | ? | N/A |  |  |
|  |  | No | 0.83 | No | Convergent/divergent validity  R VAS-pain = 0.33  R AHI helplessness = 0.36  R CES depression = 0.31  R AIMS global rating = 0.31  R Psychosocial impairment scale = 0.32  R activity limitation scale = 0.42  R VPMI active coping scale = -0.17  R VPMI passive coping scale = 0.29 | 369 |
|  |  | No | ? | N/A |  |  |
|  |  | No | ? | N/A |  |  |
|  |  |  |  |  |  |  |
| MHAQ |  | Yes | 0.90 | N/A |  | 144 |
|  |  | No | ? | N/A |  |  |
|  |  | No | PSI =  1.96 | N/A |  | 2491 |
|  |  | No | ? | N/A |  |  |
|  |  | No | ? | No | Convergent/divergent validity  R HAQ-II = 0.85  R HAQ = 0.84  R VAS-pain = 0.67  R patient assessment of global disease severity = 0.61  R VAS Fatigue = 0.55  R DAS28 = 0.50  R Physician’s global assessment of disease severity = 0.50  R stopped working = 0.35  R TJC = 0.40  R ESR = 0.22  R SJC = 0.25  R Joint surgery (Yes/no) = 0.11 | 693 |
|  |  | No | ? | N/A |  |  |
| AIMS2-SF |  | Yes | 0.87 | No | Convergent/divergent validity  R morning stiffness = 0.35  R TJC = 0.39  R SJC = 0.24 | 127 |
| ROAD  Upper extremity function |  | No | ? | Yes | Convergent/divergent validity  R SF-36 GH = -0.22  R SF-36 SF = -0.15  R SF-36 VT = -0.11  R SF-36 MH = -0.68  R SF36 BP = -0.20  R SF-36 RE = -0.17  R SF-36 RP = -0.16  R SF-36 PF = -0.18  R SF36 MCS = -0.19  R SF-36 PCS = -0.26  R HAQ = 0.29  R Patient GA functional disability = 0.25 | 159 |
|  |  | Yes | 0.89 | N/A |  | 77 |
| ROAD  Lower extremity function |  | No | ? | Yes | Convergent/divergent validity  R SF-36 GH = -0.20  R SF-36 SF = -0.17  R SF-36 VT = -0.24  R SF-36 MH = -0.91  R SF36 BP = -0.24  R SF-36 RE = -0.51  R SF-36 RP = -0.20  R SF-36 PF = -0.26  R SF36 MCS = -0.22  R SF-36 PCS = -0.26  R HAQ = 0.17  R PGA functional disability = 0.16 | 159 |
|  |  | Yes | 0.84 | N/A |  | 77 |
| ROAD  Activities of daily living |  | No | ? | Yes | Convergent/divergent validity  R SF-36 GH = -0.37  R SF-36 SF = -0.17  R SF-36 VT = -0.27  R SF-36 MH =-0.20  R SF36 BP =-0.36  R SF-36 RE = -0.16  R SF-36 RP = -0.40  R SF-36 PF = -0.29  R SF36 MCS = -0.39  R SF-36 PCS = -0.32  R HAQ = 0.32  R PGA functional disability = 0.16 | 159 |
|  |  | Yes | 0.81 | N/A |  | 77 |
| ROAD  Total score |  | No | ? | Yes | Convergent/divergent validity  R SF-36 GH = -0.23  R SF-36 SF = -0.34  R SF-36 VT = -0.30  R SF-36 MH = -0.20  R SF36 BP = -0.22  R SF-36 RE = -0.19  R SF-36 RP = -0.34  R SF-36 PF = -0.32  R SF36 MCS = -0.31  R SF-36 PCS = -0.41  R HAQ = -0.37  R PGA functional disability =-0.24 | 159 |
|  |  | No | ? | N/A |  |  |
| IRGL  Mobility |  | No | 0.93 | No | Convergent/divergent validity  R functional class = -0.55  R grip strength = 0.54  R Ritchie score = -0.54  R radiographics = -0.30  R ESR = -0.41 | 284  80  80  80  80  284 |
|  |  | No | 0.92 |  | Convergent/divergent validity  R ARA functional class = -0.49  R SJC = -0.04  R TJC= -0.20  R 30 meter walking time = -0.37  R Grip strength = 0.38  R ESR = -0.33  R CRP = -0.20  R Hb= 0.23 | 426 |
| IRGL  Self care |  | No | 0.94 | No | Convergent/divergent validity  R functional class = -0.52  R grip strength = 0.67  R Ritchie score = -0.36  R radiographics = -0.49  R ESR = -0.25 | 284  80  80  80  80  284 |
|  |  | No | 0.90 |  | Convergent/divergent validity  R ARA functional class = -0.41  R SJC = -0.09  R TJC= -0.37  R 30 meter walking time = -0.34  R Grip strength = 0.43  R ESR = -0.28  R CRP = -0.06  R Hb= 0.26 | 426 |
| FSI  Gross mobility |  | No | 0.82 | N/A |  | 149 |
| FSI  Home chores |  | No | 0.85 | N/A |  | 149 |
| FSI  Hand activities |  | No | 0.66 | N/A |  | 149 |
| FSI  Personal care |  | No | 0.88 | N/A |  | 149 |
| Short AIMS  Dexterity |  | No | 0.78 | No | Convergent/divergent validity  R joint count = 0.44  R joint alignment and motion scale = 0.41 | 180 |
| Short AIMS  Household activity | (30) | No | 0.86 | No | Convergent/divergent validity  R joint count = 0.45  R joint alignment and motion scale = 0.44 | 180 |
| Short AIMS  Activities of daily living | (30) | No | 0.63 | No | Convergent/divergent validity  R joint count = 0.37  R joint alignment and motion scale = 0.31 | 180 |
| Short AIMS  Physical activity | (30) | No | 0.74 | No | Convergent/divergent validity  R joint count = 0.37  R joint alignment and motion scale = 0.38 | 180 |
| Short AIMS  Mobility | (30) | No | 0.80 | No | Convergent/divergent validity  R joint count = 0.26  R joint alignment and motion scale = 0.29 | 180 |
| Shortened AIMS Dexterity | (32) | No | 0.80 | No | Convergent/divergent validity  R VAS-pain = 0.42  R AHI helplessness = 0.33  R CES depression = 0.35  R AIMS global rating = 0.37  R Psychosocial impairment scale = 0.30  R activity limitation scale = 0.37  R VPMI active coping scale = 0.45  R VPMI passive coping scale =0.19 | 369 |
| Shortened AIMS Household activity | (32) | No | 0.56 | No | Convergent/divergent validity  R VAS-pain = 0.38  R AHI helplessness = 0.37  R CES depression = 0.40  R AIMS global rating = 0.41  R Psychosocial impairment scale = 0.38  R activity limitation scale = 0.55  R VPMI active coping scale = -0.16  R VPMI passive coping scale = 0.29 | 369 |
| Shortened AIMS Activities of daily living | (32) | No | 0.62 | No | Convergent/divergent validity  R VAS-pain = 0.51  R AHI helplessness = 0.32  R CES depression = 0.40  R AIMS global rating = 0.29  R Psychosocial impairment scale = 0.33  R activity limitation scale = 0.40  R VPMI active coping scale = -0.11  R VPMI passive coping scale = 0.34 | 369 |
| Shortened AIMS Physical activity | (32) | No | 0.69 | No | Convergent/divergent validity  R VAS-pain = 0.44  R AHI helplessness = 0.37  R CES depression = 0.45  R AIMS global rating = 0.49  R Psychosocial impairment scale = 0.40  R activity limitation scale = 0.53  R VPMI active coping scale = -0.18  R VPMI passive coping scale = 0.23 | 369 |
| Shortened AIMS Mobility | (32) | No | 0.73 | No | Convergent/divergent validity  R VAS-pain = 0.32  R AHI helplessness = 0.34  R CES depression = 0.29  R AIMS global rating = 0.28  R Psychosocial impairment scale = 0.35  R activity limitation scale = 0.40  R VPMI active coping scale = -0.20  R VPMI passive coping scale = 0.27 | 369 |
| AIMS2  Mobility |  | Yes | 0.91 | No | Known groups validity  Scores segmented based on respondent attributing mobility level as health status problem area:  Yes: 2.42  No 0.40 | 408 |
| AIMS2  Walking and bending | (46) | Yes | 0.92 | No | Known groups validity  Scores segmented based on respondent attributing walking and bending as health status problem area:  Yes: 5.63  No: 1.47 | 408 |
| AIMS2  Hand and finger function | (46) | Yes | 0.94 | No | Known groups validity:  Scores segmented based on respondent attributing hand and finger function as health status problem area:  Yes: 3.80  No: 0.94 | 408 |
| AIMS2  Arm function | (46) | Yes | 0.92 | No | Known groups validity:  Scores segmented based on respondent attributing arm function as health status problem area:  Yes: 2.86  No: 0.51 | 408 |
| AIMS2  Self care | (46) | Yes | 0.81 | No | Known groups validity  Scores segmented based on respondent attributing self care as health status problem area:  Yes: 1.39  No: 0.23  Mean scores c with OA group | 408 |
| AIMS2  Household tasks | (46) | Yes | 0.91 | No | Known groups validity  Scores segmented based on respondent attributing household tasks as health status problem area:  Yes: 2.08  No:0.25 | 408 |
| MDHAQ |  | Yes | 0.92 | N/A |  | 144 |
| HAQ  Disability index | (36) | Yes | 0.90 | N/A |  | 144 |
|  |  | No |  | Yes | Convergent/divergent validity  R HAQ physical therapist = 0.89  R HAQ spouse = 0.87  R BDI = 0.37  R HSRD = 0.24  R VAS pain = 0.60 | 107 |
|  |  | No | ? | N/A |  |  |
|  |  | No | ? | N/A |  |  |
|  |  | No | ? | N/A |  |  |
|  |  | No | ? | N/A |  |  |
|  |  | No | ? | N/A |  |  |
|  | (37) | No | PSI = 2.37 |  |  |  |
|  |  | No | 0.88 | Yes | Known groups validity  (change scores):  Patient global:  Improve 3+ levels: -0.57  Improve 2 levels: -0.26  Improve 1 level: -0.24  Same/worse: 0.02  Physician global  Improve 3+ levels: -0.42  Improve 2 levels: -0.32  Improve 1 level: -0.17  Same/worse: -0.06  Pain assessment  Improve 3+ levels: -0.36  Improve 2 levels: -0.17  Improve 1 level: -0.22  Same/worse: 0.04  SJC  Improve 3+ levels:-0.46  Improve 2 levels: -0.27  Improve 1 level: -0.19  Same/worse: -0.03  TJC  Improve 3+ levels: -0.24  Improve 2 levels: -0.21  Improve 1 level: -0.13  Same/worse: -0.07 | 693 |
|  |  | Yes | ? | No | Convergent/divergent validity  R observer rated HAQ = 0.88  R discomfort = 0.59  R side effects = 0.20  R medical costs = 0.03 | 25 |
|  |  | No | ? | N/A |  |  |
| TFCQ |  | No | SHR = 0.66 | N/A |  | ? |
| RA-SIP |  | Yes | 0.88 | No | Convergent/divergent validity  R overall SIP = 0.47 | 99 |
|  |  |  |  |  |  |  |
|  |  |  |  |  |  |  |
|  |  |  |  |  |  |  |
|  |  |  |  |  |  |  |

* construct validity appraised for total score only

KPSS = Karnofsky Physical Status Scale

Oeh – Overall evaluation of health

Ghq-ss Somatic symptoms scale from the general health questionnaire

NHP-pm = Physical mobility subscale of the Nottingham health profile

R = Correlation coefficient

TJC = Tender joint count

SJC = Swollen joint count

RAI = Ritchie articular index

MCS = Mental component summary score of the SF-36

PCS = Physical component summary score of the SF-36

PSI = Person separation index

BDI = Beck depression inventory

HSRD= Hamilton rating scale for depression

SHR = Split half correlation

BSS= 7 item, self assessed pain scale

LAI = Lansbury articular index

CRP = C-reactive protein

ESR = Erythropoietin

HAD = Hospital anxiety and depression questionnaire

DAS-28 = Disease activity score

AHi = Arthritis helplessness index

SF-36 GH Sf-36 General health scale

SF-36 SF = Sf-36 Social functioning scale

SF-36 VT = Sf-36 Vitality scale

SF-36 MH = Sf-36 Mental health scale

SF36 BP = Sf-36 bodily pain scale

SF-36 RE = Sf-36 role emotional scale

SF-36 RP = Sf-36 role physical scale

SF-36 PF = Sf-36 physical functioning scale

VPMI = Vanderbilt pain management inventory

CES depression = Center for Epidemiologic Studies Depression Scale

Hb = hemoglobin

1. Chambers L, MacDonald L, Tugwell P, Buchanan W, Kraag G. The McMaster Health Index Questionnaire as a measure of quality of life for patients with rheumatoid disease. The Journal of Rheumatology.9(5):780.

2. Doeglas D, Krol B, Guillemin F, Suurmeijer T, Sanderman R, Smedstad L, et al. The assessment of functional status in rheumatoid arthritis: a cross cultural, longitudinal comparison of the Health Assessment Questionnaire and the Groningen Activity Restriction Scale. Journal of rheumatology. 1995;22(10):1834-43.

3. Suurmeijer T, Doeglas D, Moum T, Briançon S, Krol B, Sanderman R, et al. The Groningen Activity Restriction Scale for measuring disability: its utility in international comparisons. American Journal of Public Health. 1994;84(8):1270.

4. Baron M, Schieir O, Hudson M, Steele R, Kolahi S, Berkson L, et al. The clinimetric properties of the World Health Organization disability assessment schedule II in early inflammatory arthritis. Arthritis Care and Research. 2008;59(3):382-90.

5. Meesters JJL, Verhoef J, Liem ISL, Putter H, Vlieland TPMV. Validity and responsiveness of the World Health Organization Disability Assessment Schedule II to assess disability in rheumatoid arthritis patients. Rheumatology. 2009;49(2):326-33.

6. Bakheit AMO, Harries SR, Hull RG. Validity of a self-administered version of the Barthel Index in patients with rheumatoid arthritis. Clinical Rehabilitation. 1995;9(3):234-7.

7. Deyo RA, Inui TS, Leininger JD, Overman SS. Measuring functional outcomes in chronic disease: A comparison of traditional scales and a self-administered health status questionnaire in patients with rheumatoid arthritis. Medical Care. 1983;21(2):180-92.

8. Deyo RA, Inui TS, Leininger J, Overman S. Physical and psychosocial function in rheumatoid arthritis. Clinical use of a self-administered health status instrument. Archives of Internal Medicine. 1982;142(5):879-82.

9. Deyo RA, Inui TS. Toward clinical applications of health status measures: sensitivity of scales to clinically important changes. Health Services Research. 1984;19(3):277-89.

10. Russell AS, Conner-Spady B, Mintz A, Mallon C, Maksymowych WP. The responsiveness of generic health status measures as assessed in patients with rheumatoid arthritis receiving infliximab. Journal of rheumatology. 2003;30(5):941-7.

11. Tugwell P, Wells G, Strand V, Maetzel A, Bombardier C, Crawford B, et al. Clinical improvement as reflected in measures of function and health- related quality of life following treatment with leflunomide compared with methotrexate in patients with rheumatoid arthritis: Sensitivity and relative efficiency to detect a treatment effect in a twelve-month, placebo-controlled trial. Arthritis and Rheumatism. 2000;43(3):506-14.

12. Ruta DA, Hurst NP, Kind P, Hunter M, Stubbings A. Measuring health status in British patients with rheumatoid arthritis: Reliability, validity and responsiveness of the short form 36-item health survey (SF-36). British Journal of Rheumatology. 1998;37(4):425-36.

13. Kosinski M, Zhao SZ, Dedhiya S, Osterhaus JT, Ware Jr JE. Determining minimally important changes in generic and disease-specific health-related quality of life questionnaires in clinical trials of rheumatoid arthritis. Arthritis and Rheumatism. 2000;43(7):1478-87.

14. Birrell F, Hassell AB, Jones PW, Dawes PT. How does the short form 36 health questionnaire (SF-36) in rheumatoid arthritis (RA) relate to RA outcome measures and SF-36 population values? A cross-sectional study. Clinical Rheumatology. 2000;19(3):195-9.

15. Taylor WJ, McPherson KM. Using rasch analysis to compare the psychometric properties of the short form 36 physical function score and the health assessment questionnaire disability index in patients with psoriatic arthritis and rheumatoid arthritis. Arthritis Care and Research. 2007;57(5):723-9.

16. Tuttleman M, Pillemer SR, Tilley BC, Fowler SE, Buckley LM, Alarcón GS, et al. A cross sectional assessment of health status instruments in patients with rheumatoid arthritis participating in a clinical trial. Journal of rheumatology. 1997;24(10):1910-5.

17. Fitzpatrick R, Ziebland S, Jenkinson C, Mowat A. Importance of sensitivity to change as a criterion for selecting health status measures. Quality in health care : QHC. 1992;1(2):89-93.

18. Houssien DA, McKenna SP, Scott DL. The Nottingham Health Profile as a measure of disease activity and outcome in rheumatoid arthritis. British Journal of Rheumatology. 1997;36(1):69-73.

19. Fitzpatrick R, Ziebland S, Jenkinson C, Mowat A. A generic health status instrument in the assessment of rheumatoid arthritis. British Journal of Rheumatology. 1992;31(2):87-90.

20. Jenkinson C, Fitzpatrick R. Measurement of health status in patients with chronic illness: Comparison of the Nottingham health profile and the general health questionnaire. Family Practice. 1990;7(2):121-4.

21. Jenkinson C, Fitzpatrick R, Argyle M. The Nottingham Health Profile: An analysis of its sensitivity in differentiating illness groups. Social Science and Medicine. 1988;27(12):1411-4.

22. Egger MJ, Ward LR, Karg MB, Williams HJ, Reading JC, Alarcon GS, et al. Reliability and validity of the CSSRD Functional Assessment Survey in rheumatoid arthritis. Arthritis Care and Research. 1995;8(1):21-7.

23. Wolfe F, Michaud K, Pincus T. Development and validation of the Health Assessment Questionnaire II: A revised version of the Health Assessment Questionnaire. Arthritis and Rheumatism. 2004;50(10):3296-305.

24. Zochling J, Stucki G, Grill E, Braun J. A comparative study of patient-reported functional outcomes in acute rheumatoid arthritis. Journal of rheumatology. 2007;34(1):64-9.

25. Chiou CF, Sherbourne CD, Cornelio I, Lubeck DP, Paulus HE, Dylan M, et al. Development and validation of the revised Cedars-Sinai Health-Related Quality of Life for Rheumatoid Arthritis Instrument. Arthritis Care and Research. 2006;55(6):856-63.

26. Russak SM, Sherbourne CD, Lubeck DP, Paulus HD, Chiou CF, Sengupta N, et al. Validation of a Rheumatoid Arthritis Health-Related Quality of Life Instrument, the CSHQ-RA. Arthritis Care and Research. 2003;49(6):798-803.

27. Chiou CF, Sherbourne CD, Cornelio I, Lubeck DP, Paulus HE, Dylan M, et al. Revalidation of the original Cedars-Sinai Health-Related Quality of Life in Rheumatoid Arthritis Questionnaire. Journal of rheumatology. 2006;33(2):256-62.

28. Weisman MH, Paulus HD, Russak SM, Lubeck DP, Chiou CF, Sengupta N, et al. Development of a new instrument for rheumatoid arthritis: The Cedars-Sinai Health-Related Quality of Life instrument (CSHQ-RA). Arthritis Care and Research. 2003;49(1):78-84.

29. Meenan RF, Gertman PM, Mason JH, Dunaif R. The arthritis impact measurement scales. Further investigations of a health status measure. Arthritis and Rheumatism. 1982;25(9):1048-53.

30. Potts MK, Brandt KD. Evidence of the validity of the Arthritis Impact Measurement Scales. Arthritis and Rheumatism. 1987;30(1):93-6.

31. Lorish CD, Abraham N, Austin JS, Bradley LA, Alarcon GS. A comparison of the full and short versions of the arthritis impact measurement scales. Arthritis Care and Research. 1991;4(4):168-73.

32. Anderson JJ, Firschein HE, Meenan RF. Sensitivity of a health status measure to short-term clinical changes in arthritis. Arthritis and Rheumatism. 1989;32(7):844-50.

33. Wallston KA, Brown GK, Stein MJ, Dobbins CJ. Comparing the short and long versions of the arthritis impact measurement scales. Journal of rheumatology. 1989;16(8):1105-9.

34. Meenan RF, Anderson JJ, Kazis LE. Outcome assessment in clinical trials. Evidence for the sensitivity of a health status measure. Arthritis and Rheumatism. 1984;27(12):1344-52.

35. Buchbinder R, Bombardier C, Yeung M, Tugwell P. Which outcome measures should be used in rheumatoid arthritis clinical trials? Clinical and quality-of-life measures' responsiveness to treatment in a randomized controlled trial. Arthritis and Rheumatism. 1995;38(11):1568-80.

36. Pincus T, Sokka T, Kautiainen H. Further development of a physical function scale on a Multidimensional Health Assessment Questionnaire for standard care of patients with rheumatic diseases. Journal of Rheumatology. 2005;32(8):1432-9.

37. Wolfe F. Which HAQ is best? A comparison of the HAQ, MHAQ and RA-HAQ, a difficult 8 item HAQ (DHAQ), and a rescored 20 item HAQ (HAQ20): analyses in 2,491 rheumatoid arthritis patients following leflunomide initiation. The Journal of Rheumatology. 2001;28(5):982.

38. Ziebland S, Fitzpatrick R, Jenkinson C, Mowat A. Comparison of two approaches to measuring change in health status in rheumatoid arthritis: the Health Assessment Questionnaire (HAQ) and modified HAQ. Annals of the rheumatic diseases. 1992;51(11):1202.

39. Tugwell P, Wells G, Strand V, Maetzel A, Bombardier C, Crawford B, et al. Clinical improvement as reflected in measures of function and health-related quality of life following treatment with leflunomide compared with methotrexate in patients with rheumatoid arthritis: sensitivity and relative efficiency to detect a treatment effect in a twelve-month, placebo-controlled trial. Arthritis & Rheumatism. 2000;43(3):506-14.

40. Guillemin F, Coste J, Pouchot J, Ghézail M, Bregeon C, Sany J. The AIMS2-SF. A short form of the arthritis impact measurement scales 2. Arthritis & Rheumatism. 1997;40(7):1267-74.

41. Salaffi F, Stancati A, Neri R, Grassi W, Bombardieri S. Measuring functional disability in early rheumatoid arthritis: the validity, reliability and responsiveness of the Recent-Onset Arthritis Disability (ROAD) index. Clinical and experimental rheumatology. 2005;23(5):31.

42. Salaffi F, Bazzichi L, Stancati A, Neri R, Cazzato M, Consensi A, et al. Development of a functional disability measurement tool to assess early arthritis: the Recent-Onset Arthritis Disability (ROAD) questionnaire. Clinical and experimental rheumatology. 2005;23(5):628-36.

43. Evers A, Taal E, Kraaimaat F, Jacobs J, Abdel-Nasser A, Rasker J, et al. A comparison of two recently developed health status instruments for patients with arthritis: Dutch-AIMS2 and IRGL. Arthritis Impact Measurement Scales. Impact of Rheumatic diseases on General health and Lifestyle. Rheumatology. 1998;37(2):157.

44. Huiskes C, Kraaimaat F, Bijlsma J. Development of a self-report questionnaire to assess the impact of rheumatic diseases on health and lifestyle. J Rehabil Sci. 1990;3:65-70.

45. Jette A. Functional Status Index: reliability of a chronic disease evaluation instrument. Archives of physical medicine and rehabilitation. 1980;61(9):395.

46. Meenan R, Mason J, Anderson J, Guccione A, Kazis L. AIMS2. The content and properties of a revised and expanded Arthritis Impact Measurement Scales Health Status Questionnaire. Arthritis & Rheumatism. 1992;35(1):1-10.

47. Peck J, Smith T, Ward J, Milano R. Disability and depression in rheumatoid arthritis. A multi-trait, multi-method investigation. Arthritis & Rheumatism. 1989;32(9):1100-6.

48. Fitzpatrick R, Newman S, LAMB R, Shipley M. A comparison of measures of health status in rheumatoid arthritis. Rheumatology. 1989;28(3):201.

49. Pope JE, Khanna D, Norrie D, Ouimet JM. The minimally important difference for the health assessment questionnaire in rheumatoid arthritis clinical practice is smaller than in randomized controlled trials. Journal of rheumatology. 2009;36(2):254-9.

50. Rohekar G, Pope J. Test-Retest Reliability of Patient Global Assessment and Physician Global Assessment in Rheumatoid Arthritis. The Journal of Rheumatology. 2009;36(10):2178.

51. Bombardier C, Raboud J. A comparison of health-related quality-of-life measures for rheumatoid arthritis research*. Controlled Clinical Trials. 1991;12(4):S243-S56.

52. Fries J, Spitz P, Young D. The dimensions of health outcomes: the health assessment questionnaire, disability and pain scales. The Journal of Rheumatology. 1982;9(5):789.

53. Greenwood M, Doyle D, Ensor M. Does the Stanford Health Assessment Questionnaire have potential as a monitoring tool for subjects with rheumatoid arthritis? Annals of the rheumatic diseases. 2001;60(4):344.

54. Helewa A, Goldsmith C, Smythe H. Independent measurement of functional capacity in rheumatoid arthritis. The Journal of Rheumatology.9(5):794.

55. Sullivan M, Ahlmen M, Bjelle A, Karlsson J. Health status assessment in rheumatoid arthritis. II. Evaluation of a modified Shorter Sickness Impact Profile. The Journal of Rheumatology. 1993;20(9):1500.
