# Supplementary material for: Measurement properties of physical function scales validated for use in patients with rheumatoid arthritis: A systematic review of the literature
Source: Health Qual Life Outcomes. 2011 Nov 7;9:99. doi: 10.1186/1477-7525-9-99 (PMC3221621; doi:10.1186/1477-7525-9-99)
Supplement: Additional file 2 — Supplementary table 2: reproducibility.doc. [file 1477-7525-9-99-S2.DOC]

| Scale | Ref | Reliability | Time interval | Agreement | N |
| --- | --- | --- | --- | --- | --- |
| MHIQ  Physical function index |  | ICC = 0.53 | 1 week | ? | 30 |
| GARS  Activities of daily living |  | ? | N/A | ? | N/A |
|  |  | ? | N/A | ? | N/A |
| GARS  Instrumental activities of daily living |  | ? | N/A | ? | N/A |
|  |  | ? | N/A | ? | N/A |
| WHODAS-II  Getting around |  | ICC = 0.96 | 1 week |  | 20 |
|  |  | ? | N/A | ? | N/A |
| WHODAS-II  Self care |  | ICC = 0.82 | 1 week | ? | 20 |
|  |  | ? | N/A | ? | N/A |
| WHODAS-II  Life activities |  | ICC = 0.91 | 1 week | ? | 20 |
|  |  | ? | N/A | ? | N/A |
| Barthel index |  | ? |  | ? |  |
| SIP  Physical dimension |  | WK*=0.82 | 1-6 months | ? | 24 |
|  |  | ? |  | ? |  |
|  |  | ? |  | ? |  |
| SF-36  Physical functioning |  | ICC = 0.93 | 3 weeks | SEM = 7.16, LOA = 10.48 | 24 |
|  |  | ? |  | ? |  |
|  |  | ICC = 0.93 | 2 weeks | 2.77×within-subject standard deviation = 23.8 | 31 |
|  |  | ? |  | ? |  |
|  |  | ? |  | ? |  |
|  |  | ? |  | ? |  |
|  |  | ? |  | ? |  |
| NHP  Physical mobility |  | ? |  | ? |  |
|  |  | ? |  | ? |  |
|  |  | R = 0.78 | 3 months | ? | 73 |
|  |  |  |  |  |  |
|  |  |  |  |  |  |
| CSSRD  Personal care |  | ICC= 0.79  ICC = 0.90 | 4-6 weeks | ? | 53 |
| CSSRD  Mobility |  | ICC = 0.20  ICC = 0.50 | 4-6 weeks | ? | 53 |
| CSSRD  Transfer |  | ICC =0.64  ICC = 0.79 | 4-6 weeks | ? | 53 |
| CSSRD  Work/chores |  | ICC = 0.85  ICC = 0.91 | 4-6 weeks | ? | 53 |
| HAQ-II |  | ? |  | ? |  |
| FfbH |  | ? |  | ? |  |
| CSHQ-Ra revised Dexterity |  | ICC = 0.94 | 7-14 days | ? | 207 |
| CSHQ-Ra revised Mobility |  | ICC = 0.90 | 7-14 days | ? | 207 |
| CSHQ-RA  Dexterity |  | ICC =0.89 | 4 weeks |  | 157 |
|  |  | ICC = 0.89 |  |  |  |
|  |  | ? |  | ? |  |
| CSHQ-Ra  Mobility |  | ICC = 0.91 | 4 weeks | ? | 157 |
|  |  | ICC = 0.91 | 4 weeks | ? | 157 |
|  |  | ? |  | ? |  |
| AIMS  Dexterity |  | R = >0.80 | 2 weeks | ? | 336 |
|  |  | ? |  | ? |  |
|  |  | R = 0.53 | 6 months | ? | 155 |
|  |  | ? |  | ? |  |
|  |  | R = 0.61 | 24 months | ? | 369 |
|  |  | ? |  | ? |  |
|  |  | ? |  | ? |  |
|  |  |  |  |  |  |
|  |  |  |  |  |  |
| AIMS  Household activity |  | R = >0.80 | 2 weeks | ? | 336 |
|  |  | ? |  | ? |  |
|  |  | R = 0.68 | 6 months | ? | 155 |
|  |  | ? |  | ? |  |
|  |  | R = 0.67 | 24 months | ? | 369 |
|  |  | ? |  | ? |  |
|  |  | ? |  | ? |  |
|  |  |  |  |  |  |
| AIMS  Activities of daily living |  | R = >0.80 | 2 weeks | ? | 336 |
|  |  | ? |  | ? |  |
|  |  | R = 0.57 | 6 months | ? | 155 |
|  |  | ? |  | ? |  |
|  |  | R = 0.59 | 24 months | ? | 369 |
|  |  | ? |  | ? |  |
|  |  | ? |  | ? |  |
|  |  |  |  |  |  |
|  |  |  |  |  |  |
| AIMS  Physical activity |  | R = >0.80 | 2 weeks | ? | 336 |
|  |  | ? |  | ? |  |
|  |  | R = 0.56 | 6 months | ? | 155 |
|  |  | ? |  | ? |  |
|  |  | R = 0.62 | 24 months | ? | 369 |
|  |  | ? |  | ? |  |
|  |  | ? |  | ? |  |
|  |  |  |  |  |  |
|  |  |  |  |  |  |
| AIMS  Mobility |  | R = >0.80 | 2 weeks | ? | 336 |
|  |  | ? |  | ? |  |
|  |  | R = 0.80 | 6 months | ? | 155 |
|  |  | ? |  | ? |  |
|  |  | R = 0.57 | 24 months | ? | 369 |
|  |  | ? |  | ? |  |
|  |  | ? |  | ? |  |
| MHAQ |  | ? |  | ? |  |
|  |  | ICC = 0.90 | 3 weeks | SEM =0.14  95% LOA = 0.20 | 24 |
|  |  | ? |  | ? |  |
|  |  | ? |  | ? |  |
|  |  | ? |  | ? |  |
|  |  | ? |  | ? |  |
| AIMS2-SF |  | ICC = 0.81 | 10 days | ? | 127 |
| ROAD  Upper extremity function |  | ICC = 0.93 | 1 week | ? | 77 |
|  |  | ? |  | ? |  |
| ROAD  Lower extremity function |  | ICC = 0.89 | 1 week | ? | 77 |
|  |  | ? |  | ? |  |
| ROAD  Activities of daily living |  | ICC = 0.85 | 1 week | ? | 77 |
|  |  | ? |  | ? |  |
| ROAD  Total score |  | ? |  | ? |  |
|  |  | ? |  | ? |  |
| IRGL  Mobility |  | ? |  | ? |  |
|  |  | ? |  | ? |  |
| IRGL  Self care |  | ? |  | ? |  |
|  |  |  |  |  |  |
| FSI  Gross mobility |  | ** | 1 to 3 days | ? | 149 |
| FSI  Home chores |  | ** | 1 to 3 days | ? | 149 |
| FSI  Hand activites |  | ** | 1 to 3 days | ? | 149 |
| FSI  Personal care |  | ** | 1 to 3 days | ? | 149 |
| Short AIMS  Dexterity | (30) | R=0.53 | 6 months | ? | 155 |
| Short AIMS  Household activity | (30) | R=0.57 | 6 months | ? | 155 |
| Short AIMS  Activities of daily living | (30) | R= 0.57 | 6 months | ? | 155 |
| Short AIMS  Physical activity | (30) | R= 0.56 | 6 months | ? | 155 |
| Short AIMS  Mobility | (30) | R=0.41 | 6 months | ? | 155 |
| Shortened AIMS Dexterity | (31) | R= 0.61 | 24 months | ? | 369 |
| Shortened AIMS Household activity | (31) | R= 0.67 | 24 months | ? | 369 |
| Shortened AIMS Activities of daily living | (31) | R= 0.59 | 24 months | ? | 369 |
| Shortened AIMS Physical activity | (31) | R= 0.62 | 24 months | ? | 369 |
| Shortened AIMS Mobility | (31) | R= 0.55 | 24 months | ? | 369 |
| AIMS2  mobility |  | ICC = 0.91 | 2 weeks | ? | 45 |
| AIMS2  Walking and bending | (45) | ICC= 0.92 | 2 weeks | ? | 45 |
| AIMS2  Hand and finger function | (45) | ICC= 0.94 | 2 weeks | ? | 45 |
| AIMS2  Arm function | (45) | ICC= 0.92 | 2 weeks | ? | 45 |
| AIMS2  Self care | (45) | ICC= 0.81 | 2 weeks | ? | 45 |
| AIMS2  Household tasks | (45) | ICC= 0.91 | 2 weeks | ? | 45 |
| MDHAQ |  | ? |  | ? |  |
| HAQ  Disability index | (35) |  |  |  |  |
|  |  | ? |  | ? |  |
|  |  | ? |  | ? |  |
|  |  | ? |  | ? |  |
|  |  | ICC = 0.90 | 1-3 days | ? | 122 |
|  |  |  |  |  |  |
|  |  |  |  |  |  |
|  | (36) |  |  |  |  |
|  | (12) |  |  |  |  |
|  |  |  |  |  |  |
|  |  |  | 2 months | ***95% LOA = 0.48 | 45 |
|  |  | ? |  | ? |  |
|  |  | ? |  | ? |  |

* Wk = weighted kappa

** ICC for individual items

*** LOA = limits of agreement

1. Chambers L, MacDonald L, Tugwell P, Buchanan W, Kraag G. The McMaster Health Index Questionnaire as a measure of quality of life for patients with rheumatoid disease. The Journal of Rheumatology.9(5):780.

2. Doeglas D, Krol B, Guillemin F, Suurmeijer T, Sanderman R, Smedstad L, et al. The assessment of functional status in rheumatoid arthritis: a cross cultural, longitudinal comparison of the Health Assessment Questionnaire and the Groningen Activity Restriction Scale. Journal of rheumatology. 1995;22(10):1834-43.

3. Suurmeijer T, Doeglas D, Moum T, Briançon S, Krol B, Sanderman R, et al. The Groningen Activity Restriction Scale for measuring disability: its utility in international comparisons. American Journal of Public Health. 1994;84(8):1270.

4. Baron M, Schieir O, Hudson M, Steele R, Kolahi S, Berkson L, et al. The clinimetric properties of the World Health Organization disability assessment schedule II in early inflammatory arthritis. Arthritis Care and Research. 2008;59(3):382-90.

5. Meesters JJL, Verhoef J, Liem ISL, Putter H, Vlieland TPMV. Validity and responsiveness of the World Health Organization Disability Assessment Schedule II to assess disability in rheumatoid arthritis patients. Rheumatology. 2009;49(2):326-33.

6. Bakheit AMO, Harries SR, Hull RG. Validity of a self-administered version of the Barthel Index in patients with rheumatoid arthritis. Clinical Rehabilitation. 1995;9(3):234-7.

7. Deyo RA, Inui TS, Leininger JD, Overman SS. Measuring functional outcomes in chronic disease: A comparison of traditional scales and a self-administered health status questionnaire in patients with rheumatoid arthritis. Medical Care. 1983;21(2):180-92.

8. Deyo RA, Inui TS, Leininger J, Overman S. Physical and psychosocial function in rheumatoid arthritis. Clinical use of a self-administered health status instrument. Archives of Internal Medicine. 1982;142(5):879-82.

9. Deyo RA, Inui TS. Toward clinical applications of health status measures: sensitivity of scales to clinically important changes. Health Services Research. 1984;19(3):277-89.

10. Russell AS, Conner-Spady B, Mintz A, Mallon C, Maksymowych WP. The responsiveness of generic health status measures as assessed in patients with rheumatoid arthritis receiving infliximab. Journal of rheumatology. 2003;30(5):941-7.

11. Tugwell P, Wells G, Strand V, Maetzel A, Bombardier C, Crawford B, et al. Clinical improvement as reflected in measures of function and health- related quality of life following treatment with leflunomide compared with methotrexate in patients with rheumatoid arthritis: Sensitivity and relative efficiency to detect a treatment effect in a twelve-month, placebo-controlled trial. Arthritis and Rheumatism. 2000;43(3):506-14.

12. Ruta DA, Hurst NP, Kind P, Hunter M, Stubbings A. Measuring health status in British patients with rheumatoid arthritis: Reliability, validity and responsiveness of the short form 36-item health survey (SF-36). British Journal of Rheumatology. 1998;37(4):425-36.

13. Kosinski M, Zhao SZ, Dedhiya S, Osterhaus JT, Ware Jr JE. Determining minimally important changes in generic and disease-specific health-related quality of life questionnaires in clinical trials of rheumatoid arthritis. Arthritis and Rheumatism. 2000;43(7):1478-87.

14. Birrell F, Hassell AB, Jones PW, Dawes PT. How does the short form 36 health questionnaire (SF-36) in rheumatoid arthritis (RA) relate to RA outcome measures and SF-36 population values? A cross-sectional study. Clinical Rheumatology. 2000;19(3):195-9.

15. Taylor WJ, McPherson KM. Using rasch analysis to compare the psychometric properties of the short form 36 physical function score and the health assessment questionnaire disability index in patients with psoriatic arthritis and rheumatoid arthritis. Arthritis Care and Research. 2007;57(5):723-9.

16. Tuttleman M, Pillemer SR, Tilley BC, Fowler SE, Buckley LM, Alarcón GS, et al. A cross sectional assessment of health status instruments in patients with rheumatoid arthritis participating in a clinical trial. Journal of rheumatology. 1997;24(10):1910-5.

17. Fitzpatrick R, Ziebland S, Jenkinson C, Mowat A. Importance of sensitivity to change as a criterion for selecting health status measures. Quality in health care : QHC. 1992;1(2):89-93.

18. Houssien DA, McKenna SP, Scott DL. The Nottingham Health Profile as a measure of disease activity and outcome in rheumatoid arthritis. British Journal of Rheumatology. 1997;36(1):69-73.

19. Fitzpatrick R, Ziebland S, Jenkinson C, Mowat A. A generic health status instrument in the assessment of rheumatoid arthritis. British Journal of Rheumatology. 1992;31(2):87-90.

20. Jenkinson C, Fitzpatrick R. Measurement of health status in patients with chronic illness: Comparison of the Nottingham health profile and the general health questionnaire. Family Practice. 1990;7(2):121-4.

21. Jenkinson C, Fitzpatrick R, Argyle M. The Nottingham Health Profile: An analysis of its sensitivity in differentiating illness groups. Social Science and Medicine. 1988;27(12):1411-4.

22. Egger MJ, Ward LR, Karg MB, Williams HJ, Reading JC, Alarcon GS, et al. Reliability and validity of the CSSRD Functional Assessment Survey in rheumatoid arthritis. Arthritis Care and Research. 1995;8(1):21-7.

23. Wolfe F, Michaud K, Pincus T. Development and validation of the Health Assessment Questionnaire II: A revised version of the Health Assessment Questionnaire. Arthritis and Rheumatism. 2004;50(10):3296-305.

24. Zochling J, Stucki G, Grill E, Braun J. A comparative study of patient-reported functional outcomes in acute rheumatoid arthritis. Journal of rheumatology. 2007;34(1):64-9.

25. Chiou CF, Sherbourne CD, Cornelio I, Lubeck DP, Paulus HE, Dylan M, et al. Development and validation of the revised Cedars-Sinai Health-Related Quality of Life for Rheumatoid Arthritis Instrument. Arthritis Care and Research. 2006;55(6):856-63.

26. Russak SM, Sherbourne CD, Lubeck DP, Paulus HD, Chiou CF, Sengupta N, et al. Validation of a Rheumatoid Arthritis Health-Related Quality of Life Instrument, the CSHQ-RA. Arthritis Care and Research. 2003;49(6):798-803.

27. Chiou CF, Sherbourne CD, Cornelio I, Lubeck DP, Paulus HE, Dylan M, et al. Revalidation of the original Cedars-Sinai Health-Related Quality of Life in Rheumatoid Arthritis Questionnaire. Journal of rheumatology. 2006;33(2):256-62.

28. Weisman MH, Paulus HD, Russak SM, Lubeck DP, Chiou CF, Sengupta N, et al. Development of a new instrument for rheumatoid arthritis: The Cedars-Sinai Health-Related Quality of Life instrument (CSHQ-RA). Arthritis Care and Research. 2003;49(1):78-84.

29. Meenan RF, Gertman PM, Mason JH, Dunaif R. The arthritis impact measurement scales. Further investigations of a health status measure. Arthritis and Rheumatism. 1982;25(9):1048-53.

30. Potts MK, Brandt KD. Evidence of the validity of the Arthritis Impact Measurement Scales. Arthritis and Rheumatism. 1987;30(1):93-6.

31. Lorish CD, Abraham N, Austin JS, Bradley LA, Alarcon GS. A comparison of the full and short versions of the arthritis impact measurement scales. Arthritis Care and Research. 1991;4(4):168-73.

32. Anderson JJ, Firschein HE, Meenan RF. Sensitivity of a health status measure to short-term clinical changes in arthritis. Arthritis and Rheumatism. 1989;32(7):844-50.

33. Wallston KA, Brown GK, Stein MJ, Dobbins CJ. Comparing the short and long versions of the arthritis impact measurement scales. Journal of rheumatology. 1989;16(8):1105-9.

34. Meenan RF, Anderson JJ, Kazis LE. Outcome assessment in clinical trials. Evidence for the sensitivity of a health status measure. Arthritis and Rheumatism. 1984;27(12):1344-52.

35. Buchbinder R, Bombardier C, Yeung M, Tugwell P. Which outcome measures should be used in rheumatoid arthritis clinical trials? Clinical and quality-of-life measures' responsiveness to treatment in a randomized controlled trial. Arthritis and Rheumatism. 1995;38(11):1568-80.

36. Pincus T, Sokka T, Kautiainen H. Further development of a physical function scale on a Multidimensional Health Assessment Questionnaire for standard care of patients with rheumatic diseases. Journal of Rheumatology. 2005;32(8):1432-9.

37. Wolfe F. Which HAQ is best? A comparison of the HAQ, MHAQ and RA-HAQ, a difficult 8 item HAQ (DHAQ), and a rescored 20 item HAQ (HAQ20): analyses in 2,491 rheumatoid arthritis patients following leflunomide initiation. The Journal of Rheumatology. 2001;28(5):982.

38. Ziebland S, Fitzpatrick R, Jenkinson C, Mowat A. Comparison of two approaches to measuring change in health status in rheumatoid arthritis: the Health Assessment Questionnaire (HAQ) and modified HAQ. Annals of the rheumatic diseases. 1992;51(11):1202.

39. Tugwell P, Wells G, Strand V, Maetzel A, Bombardier C, Crawford B, et al. Clinical improvement as reflected in measures of function and health-related quality of life following treatment with leflunomide compared with methotrexate in patients with rheumatoid arthritis: sensitivity and relative efficiency to detect a treatment effect in a twelve-month, placebo-controlled trial. Arthritis & Rheumatism. 2000;43(3):506-14.

40. Guillemin F, Coste J, Pouchot J, Ghézail M, Bregeon C, Sany J. The AIMS2-SF. A short form of the arthritis impact measurement scales 2. Arthritis & Rheumatism. 1997;40(7):1267-74.

41. Salaffi F, Stancati A, Neri R, Grassi W, Bombardieri S. Measuring functional disability in early rheumatoid arthritis: the validity, reliability and responsiveness of the Recent-Onset Arthritis Disability (ROAD) index. Clinical and experimental rheumatology. 2005;23(5):31.

42. Salaffi F, Bazzichi L, Stancati A, Neri R, Cazzato M, Consensi A, et al. Development of a functional disability measurement tool to assess early arthritis: the Recent-Onset Arthritis Disability (ROAD) questionnaire. Clinical and experimental rheumatology. 2005;23(5):628-36.

43. Evers A, Taal E, Kraaimaat F, Jacobs J, Abdel-Nasser A, Rasker J, et al. A comparison of two recently developed health status instruments for patients with arthritis: Dutch-AIMS2 and IRGL. Arthritis Impact Measurement Scales. Impact of Rheumatic diseases on General health and Lifestyle. Rheumatology. 1998;37(2):157.

44. Huiskes C, Kraaimaat F, Bijlsma J. Development of a self-report questionnaire to assess the impact of rheumatic diseases on health and lifestyle. J Rehabil Sci. 1990;3:65-70.

45. Jette A. Functional Status Index: reliability of a chronic disease evaluation instrument. Archives of physical medicine and rehabilitation. 1980;61(9):395.

46. Meenan R, Mason J, Anderson J, Guccione A, Kazis L. AIMS2. The content and properties of a revised and expanded Arthritis Impact Measurement Scales Health Status Questionnaire. Arthritis & Rheumatism. 1992;35(1):1-10.

47. Peck J, Smith T, Ward J, Milano R. Disability and depression in rheumatoid arthritis. A multi-trait, multi-method investigation. Arthritis & Rheumatism. 1989;32(9):1100-6.

48. Fitzpatrick R, Newman S, LAMB R, Shipley M. A comparison of measures of health status in rheumatoid arthritis. Rheumatology. 1989;28(3):201.

49. Pope JE, Khanna D, Norrie D, Ouimet JM. The minimally important difference for the health assessment questionnaire in rheumatoid arthritis clinical practice is smaller than in randomized controlled trials. Journal of rheumatology. 2009;36(2):254-9.

50. Rohekar G, Pope J. Test-Retest Reliability of Patient Global Assessment and Physician Global Assessment in Rheumatoid Arthritis. The Journal of Rheumatology. 2009;36(10):2178.

51. Bombardier C, Raboud J. A comparison of health-related quality-of-life measures for rheumatoid arthritis research*. Controlled Clinical Trials. 1991;12(4):S243-S56.

52. Fries J, Spitz P, Young D. The dimensions of health outcomes: the health assessment questionnaire, disability and pain scales. The Journal of Rheumatology. 1982;9(5):789.

53. Greenwood M, Doyle D, Ensor M. Does the Stanford Health Assessment Questionnaire have potential as a monitoring tool for subjects with rheumatoid arthritis? Annals of the rheumatic diseases. 2001;60(4):344.

54. Helewa A, Goldsmith C, Smythe H. Independent measurement of functional capacity in rheumatoid arthritis. The Journal of Rheumatology.9(5):794.

55. Sullivan M, Ahlmen M, Bjelle A, Karlsson J. Health status assessment in rheumatoid arthritis. II. Evaluation of a modified Shorter Sickness Impact Profile. The Journal of Rheumatology. 1993;20(9):1500.
